# In-plane oxygen diffusion measurements in polymer films using time-resolved imaging of programmable luminescent tags

**DOI:** 10.1038/s41598-024-56237-5

**Published:** 2024-03-09

**Authors:** Richard Kantelberg, Tim Achenbach, Anton Kirch, Sebastian Reineke

**Affiliations:** 1 Dresden Integrated Center for Applied Physics and Photonic Materials (IAPP) and Institute of Applied Physics, Technische Universität Dresden, Nöthnitzer Straße 61, 01187 Dresden, Germany; 2 The Organic Photonics and Electronics Group, Department of Physics, Umeå University, 90187 Umeå, Sweden

**Keywords:** Information storage, Optical materials and structures, Electronic properties and materials

## Abstract

Oxygen diffusion properties in thin polymer films are key parameters in industrial applications from food packaging, over medical encapsulation to organic semiconductor devices and have been continuously investigated in recent decades. The established methods have in common that they require complex pressure-sensitive setups or vacuum technology and usually do not come without surface effects. In contrast, this work provides a low-cost, precise and reliable method to determine the oxygen diffusion coefficient *D* in bulk polymer films based on tracking the phosphorescent pattern of a programmable luminescent tag over time. Our method exploits two-dimensional image analysis of oxygen-quenched organic room-temperature phosphors in a host polymer with high spatial accuracy. It avoids interface effects and accounts for the photoconsumption of oxygen. As a role model, the diffusion coefficients of polystyrene glasses with molecular weights between 13k and 350k g/mol are determined to be in the range of (0.8–1.5) × 10^–7^ cm^2^/s, which is in good agreement with previously reported values. We finally demonstrate the reduction of the oxygen diffusion coefficient in polystyrene by one quarter upon annealing above its glass transition temperature.

## Introduction

**Figure 1:**
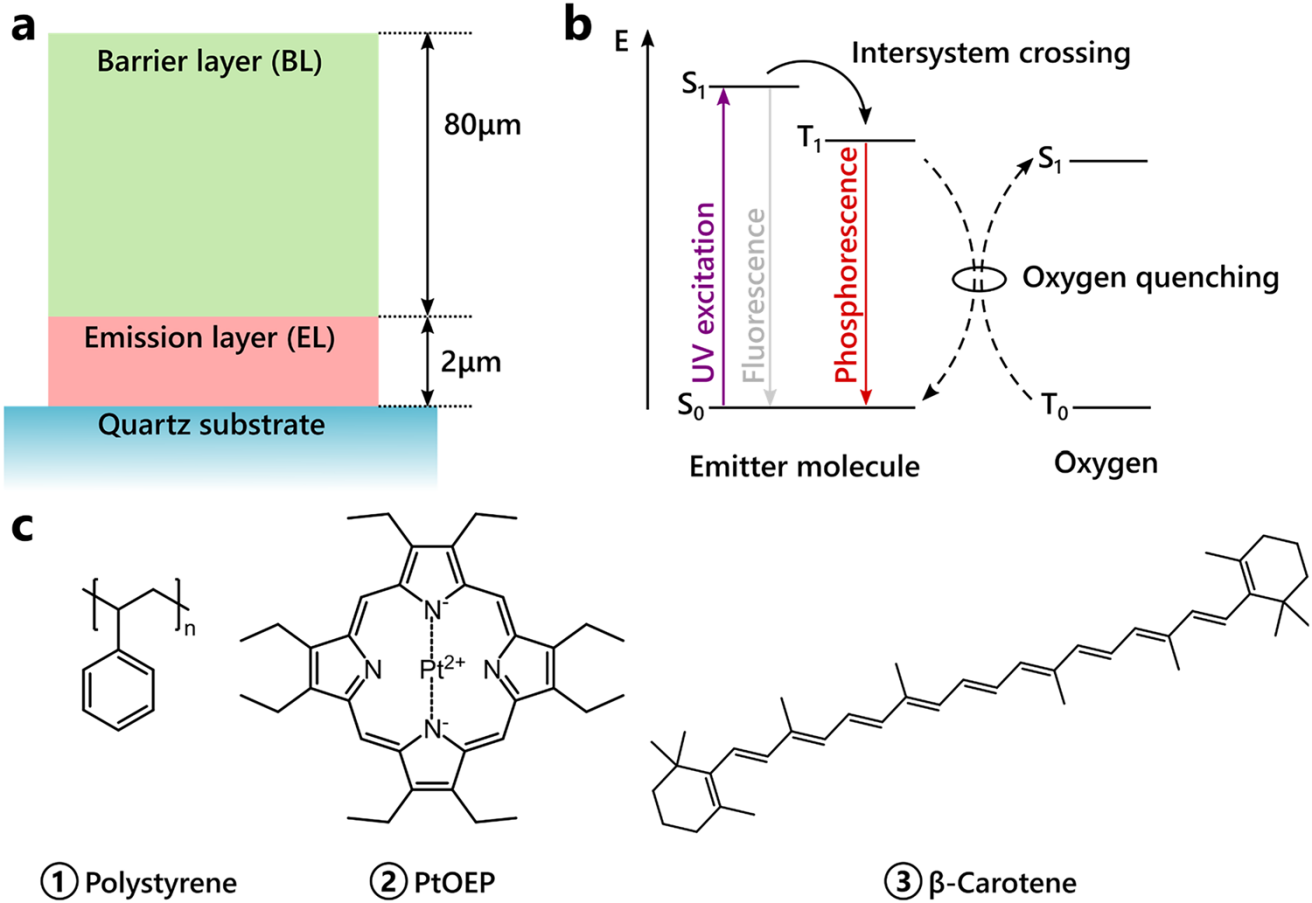
(**a**) Film design: the emission layer is spin cast onto a quartz glass substrate and covered with an oxygen barrier (Exceval™) by drop casting. (**b**) Schematic of the mechanism of luminescence and oxygen quenching in the emission layer: upon UV excitation, excited singlet states are generated, which almost exclusively convert into triplet states by intersystem crossing. These triplets can transition back to the ground state radiatively or non-radiatively. In the presence of oxygen, they may be quenched and excited singlet oxygen is generated. (**c**) Chemical structures of the materials used in the emission layer.

Diffusion properties of oxygen in thin polymer films are of pivotal importance in the vast majority of state-of-the-art applications that utilize modern functional materials. Owing to their versatile character, polymers are for example applied in food packaging^1^, medicine, such as contact lenses or encapsulation of antibiotics^2^, and organic semiconducting devices, like organic light-emitting diodes, photovoltaics^3^, or sensors^4^. As oxygen itself is not easy to measure, many sensing techniques rely on its property of quenching excited states of luminescent materials^5^. Figure 1b introduces this concept, where the triplet ground state of oxygen gets excited via an energy transfer-mediated deactivation of the luminophore’s excited triplet state^6^.

Methods for characterizing oxygen diffusion in and through polymers using luminescence quenching have been known for more than half a century^7,8^ and have found their way into commercially available oxygen sensors^9^. Quenching based techniques mostly rely on either collisional fluorescence quenching^10^, non-radiative phosphorescence quenching by electronic excitation of oxygen molecules^11,12^, or singlet oxygen emission^13^. However, the usual methods have in common to require expensive and heavy machinery such as vacuum pumps and gas mixing setups^14–19^.

The method presented in this work is based on tracking the temporal evolution of an organic bilayer system, previously reported as a programmable luminescent tag (PLT)^20–22^. As shown in Fig. 1a, it consists of a doped polymer film (emission layer) and an oxygen-impermeable covering (barrier layer). This concept can be used in many different industrial settings, ranging from (in)visible package labeling over UV sensing to security features, depending on the doping species^21^. To study the in-plane oxygen diffusion within a PLT, PtOEP as a dye and beta-carotene as an antioxidant are doped into a polystyrene host polymer (chemical structures Fig. 1c), forming a well-functioning emission layer system.

The oxygen concentration in the film can be deliberately depleted by UV treatment in a spatially defined manner using a shadow mask. A camera can then be used to image the imprinted pattern over time, which blurs as oxygen diffuses back into the initially depleted areas. By applying the well-established Stern–Volmer equation, the oxygen concentration can be calculated and monitored over time. This method can be used to model the spatially resolved oxygen concentration and extract the diffusion coefficient *D*.

## Experimental approach

Our motivation for developing a measurement technique for oxygen sensing in bulk polymer films is to find a method that uses the simplest possible setup, can be conducted contactless, and does not require vacuum machinery.

The film under investigation comprises polystyrene doped with the oxygen-sensitive phosphorescent dye platinum-octaethyl-porphyrin (PtOEP) and a singlet oxygen trapping molecule (beta-carotene). PtOEP is a common dye for optical oxygen sensing. For our experiment it seems to be a suitable trade-off for resolving both low and high oxygen concentrations due its triplet state lifetime of about 50 microseconds^21^.

Beta-carotene acts as an antioxidant and protects the emitter molecule from fast degradation at high oxygen concentrations. The host–guest blend is spin cast from solution onto a simple quartz glass substrate. To protect it from vertical oxygen penetration, an oxygen barrier layer consisting of Exceval™ was finally added via drop casting, cf. Fig. 1a.

**Figure 2:**
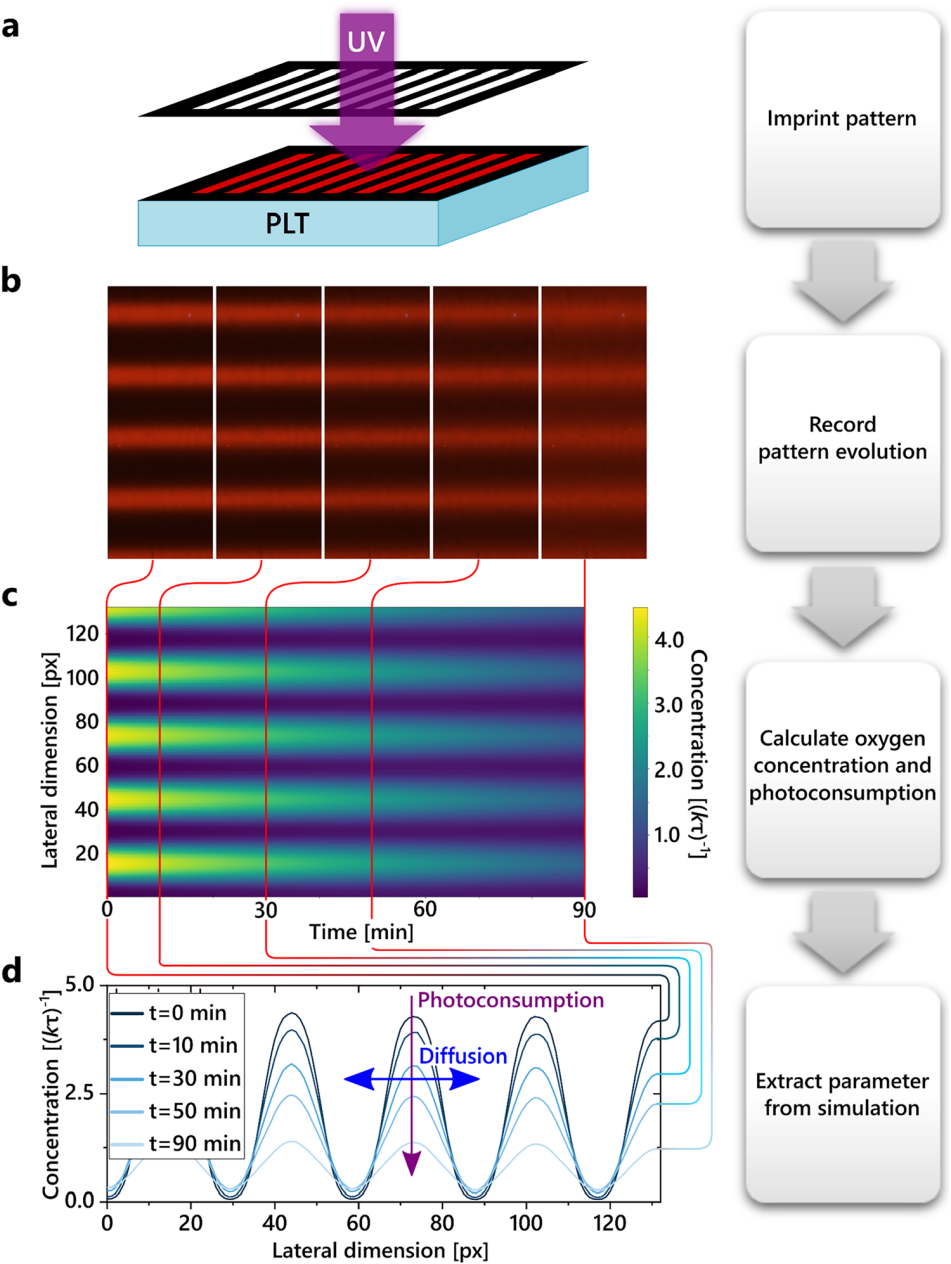
Flowchart of the data acquisition routine: (**a**) using a UV source and a shadow mask, a luminescent pattern is imprinted into the active film. (**b**) The pattern is recorded over time with a camera. (**c**) The spatial oxygen concentration distribution over time is calculated from the image series and (**d**) the diffusion coefficient *D* is extracted.

Figure 2 presents the general experimental methodology including the important steps for data acquisition. With a shadow mask and a UV LED, we create a deterministic spatial oxygen concentration gradient. This effect is accomplished by the photoconsumption^23,24^ of molecular oxygen at the illuminated spots, cf. Fig. 2a. After removing the shadow mask, the oxygen gradients can be observed as a striped pattern. The sample areas treated with UV light are depleted of oxygen and exhibit RTP under excitation. This can be identified as bright red stripes in Fig. 2b. Subsequently, due to the induced concentration gradient, the oxygen will diffuse back into the depleted regions. This diffusion process smears out the imprinted pattern and can be monitored using a top-mounted CCD camera. Recording the local intensity pattern of the oxygen-quenched phosphorescence over time, as indicated in Fig. 2b,c, allows us to spatiotemporally calculate the oxygen concentration evolution in the film. Therefore, we use the linear approximation of the Stern–Volmer equation^25^

1 $$I\left(\left[Q\right]\right)=\frac{I\left(\left[Q\right]=0\right)}{1+{k}_\text{q} {\tau }_{0}\left[Q\right]} ,$$

with *I*([*Q*]) being the phosphorescence intensity, *k*_q_ the oxygen quenching constant, *τ*_0_ the emitter triplet state lifetime in the absence of oxygen (*τ*_0_ = *τ*([*Q*] = 0)), and [*Q*] the oxygen concentration.

Since we know both the time characteristics of the quenching process and the respective UV dose for each measurement, we can determine the share of photoconsumption on the total change in oxygen concentration. Finally, as indicated in Fig. 2d, we fit a diffusion simulation of the initial concentration, which also subtracts the calculated share of photoconsumption, to find the value of the diffusion coefficient in the polymer film. The diffusion simulation is based on the inhomogeneous heat equation (cf. Eq. (2)) with initial and boundary conditions given by the experiment and the photoconsumption representing the inhomogeneity. To accurately model the diffusion behaviour, a detailed understanding of how the CCD camera recordings translate to the mathematical description of the diffusion process is key. This is explained briefly in the following section on data evaluation and in detail in “Methods” section.

## Data evaluation

**Figure 3:**
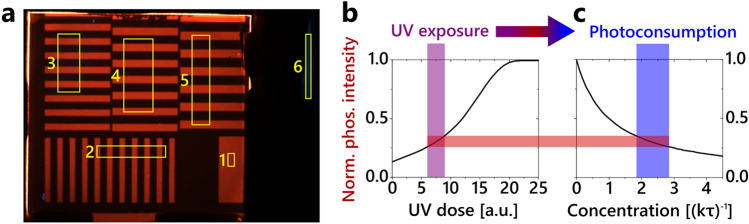
(**a**) Image of the imprinted luminescent pattern showing the positions where the sample is evaluated (spots 1–5). Spot 6 indicates the position of the LED intensity tracker. (**b**) Reference activation curve of the system. It shows the normalized phosphorescence intensity of the film as a function of the UV dose to which it is exposed. (**c**) The detected phosphorescence intensity can be related to the oxygen concentration using the Stern–Volmer equation. The decreased amount of oxygen due to photoconsumption (blue bar) can now be related to the UV exposure (purple bar).

Before starting the data acquisition process, we take an image of a sample completely depleted of oxygen. This sets an upper limit to the expected luminescence intensity and the exposure time of the camera is adjusted so that the camera does not run into saturation during the measurement. Starting with the imprinting of the stripe pattern via a shadow mask, the series of images is recorded. An exemplary image of the resulting red luminescent pattern is displayed in Fig. 3a. All recorded images are normalized to their respective LED intensity, tracked in spot 6, and the last image showing the fully activated sample. From the red pixel values of the acquired data, we extract the reference activation curve, i.e., the strictly monotonic increase of an unpatterned and unperturbed region on the sample (spot 1) due to oxygen photoconsumption only, cf. Fig. 3b. Next, the areas to be evaluated for the diffusion measurement are defined, labelled as spots 2–5 in Fig. 3a. Each spot covers an area of approximately 10–25 mm^2^ and is resolved by about 62 µm per pixel dimension. The grid spacing of the individual areas 2–5 varies in ascending order from 0.7 to 1.0 mm at intervals of 0.1 mm.

Each region of interest is averaged along the short dimension parallel to the striped profile to reduce the data sets to one spatial dimension. From this, the oxygen concentration [*Q*] and photoconsumption [*Q*]_PC_ are calculated. Each value at each time step in the 1D intensity profile is interpreted according to the following routine: first, the intensity is assigned to the interpolated reference activation curve shown in Fig. 3b. Second, the intensity changes during imaging are taken into account, as each read-out cycle induces some photoconsumption of oxygen, cf. Fig. 3c. The recorded image gives the integrated intensity over the imaging period. Knowing the UV dose exposed to the film, we can trace back to the intensity state prior and posterior to the illumination. Third, the oxygen concentration of the prior and posterior intensity states is calculated using the Stern–Volmer equation given in Eq. (1), rearranged to [*Q*]. The difference between the prior and posterior concentrations gives the amount of oxygen photoconsumed during the imaging process. Fourth, we simulate the oxygen diffusion using the inhomogeneous heat equation

2 $$\frac{\partial }{\partial t}[Q]=D\frac{{\partial }^{2}}{\partial {x}^{2}}[Q]+F{[Q}_{\text{PC}}],$$

where [*Q*] is the oxygen concentration, *D* the oxygen diffusion coefficient, [*Q*_PC_] the photoconsumed oxygen concentration, and *F* the photoconsumption scaling factor. The latter accounts for the spatial inhomogeneity of the LED intensity and can be interpreted as a stretching (*F* < 1) or compression (*F* > 1) of the reference activation curve with respect to the measurement position. From a mathematical point of view, the units of the concentration cancel out when resolving the heat differential equation. Consequently, relative concentration units such as (*kτ*)^−1^ are sufficient for determining the diffusion constant. As a result, the presented method can be applied even without a thorough knowledge of the absolute partial pressure, which eases measurement and data evaluation significantly.

We choose the posterior concentration as initial condition and as boundary conditions and the calculated oxygen photoconsumption as inhomogeneity. Using the simulation, we fit two parameters in order to match the experimental data: first, the oxygen diffusion coefficient *D* and second, the photoconsumption scaling factor *F*.

## Results

**Figure 4:**
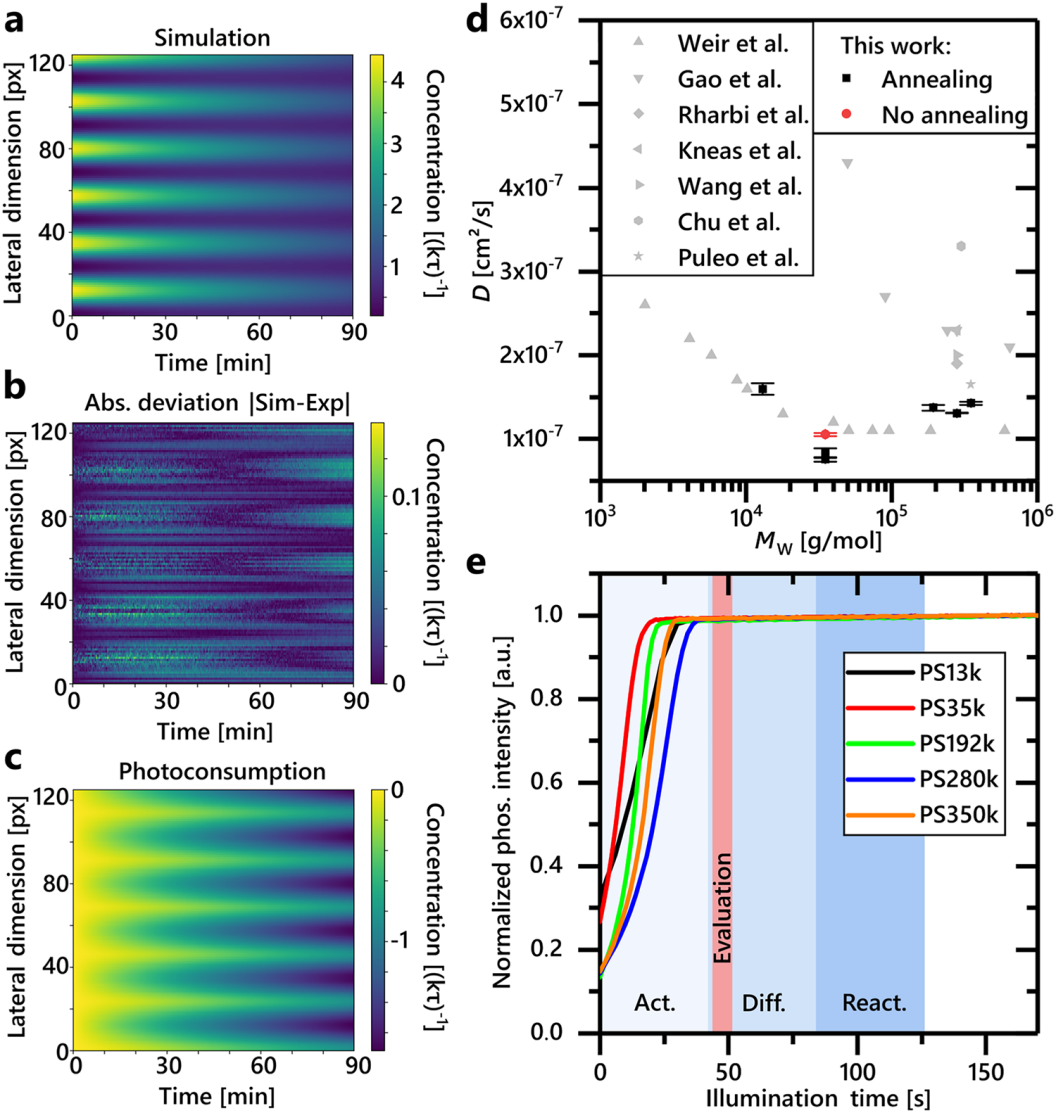
(**a**) The simulation result of PS280k shows the temporal evolution of the striped oxygen concentration pattern, reduced to one spatial dimension. (**b**) Absolute deviation of the simulation in (**a**) and the experimental recorded data. The maximum relative deviation is less than 3%. (**c**) Integrated photoconsumption during the evaluated time period. In non-activated regions of the pattern, about 45% of the molecular oxygen is removed by photoconsumption. (**d**) The diffusion coefficient *D* of polystyrene in dependency of the molecular weight *M*_W_. Our results fit the general trend of literature values (references: Weir^26^, Gao et al.^27,28^, Rharbi^29^, Kneas^30^, Wang^31^, Chu^32^, Puleo^15^). Furthermore, for PS35k, a difference in the resulting diffusion coefficient can be observed between samples annealed at 115 °C for 15 min and non-annealed samples. (**e**) Shown is the normalized phosphorescence intensity as a function of illumination time in our measurement setup. The sample shown here is homogeneously activated in space without any shadow mask pattern, i.e., with no lateral diffusion. The illumination of each sample follows a duty cycle of 10–40 ms of active illumination followed by a 15 min pause. The pause time is excluded from the diagram. The total time of the depicted process spans about 12.5 h, respectively. The blue background shading represents the three phases of the measurement with respect to the total illumination time (activation, diffusion, reactivation). The later evaluated time period is indicated by the red shading. No sample shows any degradation during the measurement.

Here, we apply our new method to measure the diffusion coefficient *D* in 5 different polystyrene glasses with molecular weights ranging from 13k to 350k. The determined values for *D* range between (0.76–1.60) × 10^–7^ cm^2^/s, as shown in the overview in Fig. 4d and Table 1, depending on the polymer chain length. The values can be provided with an accuracy of ± 7.2% in the worst case and ± 0.3% in the best case, depending on the spatial homogeneity of the sample. With the exception of PS35k, the resulting *D* values are in good agreement with the general trend of previously published data. However, no clear trend of *D* scaling with the molecular weight is observed in the range tested here.

**Table 1:** Measured diffusion coefficients *D* for polystyrene samples with different molecular weight *M*_W_*.*

Molecular weight	Annealing	Diffusion coefficient	Measurement uncertainty	Relative uncertainty
*M*_W_ (g/mol)	Yes/no	*D *(10^–7^ cm^2^/s)	Δ*D *(10^–7^ cm^2^/s)	$\frac{\Delta D}{D}$ (%)
13,000	Yes	1.60	0.07	4.4
35,000	Yes	0.83	0.06	7.2
35,000	Yes	0.756	0.024	3.2
35,000	No	1.056	0.019	1.8
192,000	Yes	1.37	0.04	2.9
280,000	Yes	1.311	0.004	0.3
350,000	Yes	1.429	0.018	1.3

We employed beta-carotene as an antioxidant to prevent the sample's phosphorescent emitter from rapid photodegradation by reaction with singlet oxygen. We expect the protection mechanism to be both quenching of ^1^O_2_ by beta-carotene and oxidation of beta-carotene by endoperoxide formation^38,39^. Figure 4e shows the activation of the phosphorescence for all polymers to its maximum intensity and an additional illumination for over 120 s. This far exceeds the total illumination time during a diffusion measurement. It can therefore be assumed that the dopant mixture effectively protects the emitter and prevents photodegradation for the UV exposure times applied in the presented experiments. The three corresponding measurement phases of activation, diffusion and reactivation are indicated by the blue shading. The red shaded area represents the time period used later for data evaluation.

Figure 4a–c depict that the simulation based on the inhomogeneous heat equation with the calculated amount of photoconsumed molecular oxygen and the experimental data as initial and boundary conditions seems to model the behaviour accurately. Figure 4a shows the time evolution of the simulated concentration. The stripe pattern is very prominent in the concentration level of the initial state. High concentrations are representing dark areas on the sample and vice versa. Due to diffusion and photoconsumption, the concentration gradient fades out over time, i.e. molecular oxygen diffuses from the dark areas on the sample into the light shaded areas of the sample. It can be seen in Fig. 4b that the absolute deviation between the experiment and the fitted simulation is comparably small. The difference between the prior and the posterior oxygen concentration represents the photoconsumption at each time step. The time-integrated photoconsumption in Fig. 4c shows how much total oxygen is removed from the emission layer during the measurement until the respective time. It is small at the beginning of the measurement and plays a prominent role in the regions of high initial oxygen concentrations, i.e. the dark regions of the sample. Its magnitude is a significant share of the maximum oxygen concentration.

Annealing the samples for 15 min at 115 °C, which is above the glass transition temperature of the polymers used (*T*_g_ ≈ 100 °C), significantly alters the diffusion coefficient as shown in Fig. 4d. A decrease in *D* from 1.06 × 10^–7^ to 0.80 × 10^–7^ cm^2^/s for annealed samples can be observed for PS35k. This is in agreement with our expectation, since the annealing process reduces the thickness of the emission layer of PS35k samples from 8440 ± 60 nm to 7420 ± 60 nm, which is approximately 12%. As a result, the density of the layer increases, which should lead to a decrease of the free volume and thus a lower diffusion coefficient.

**Figure 5:**
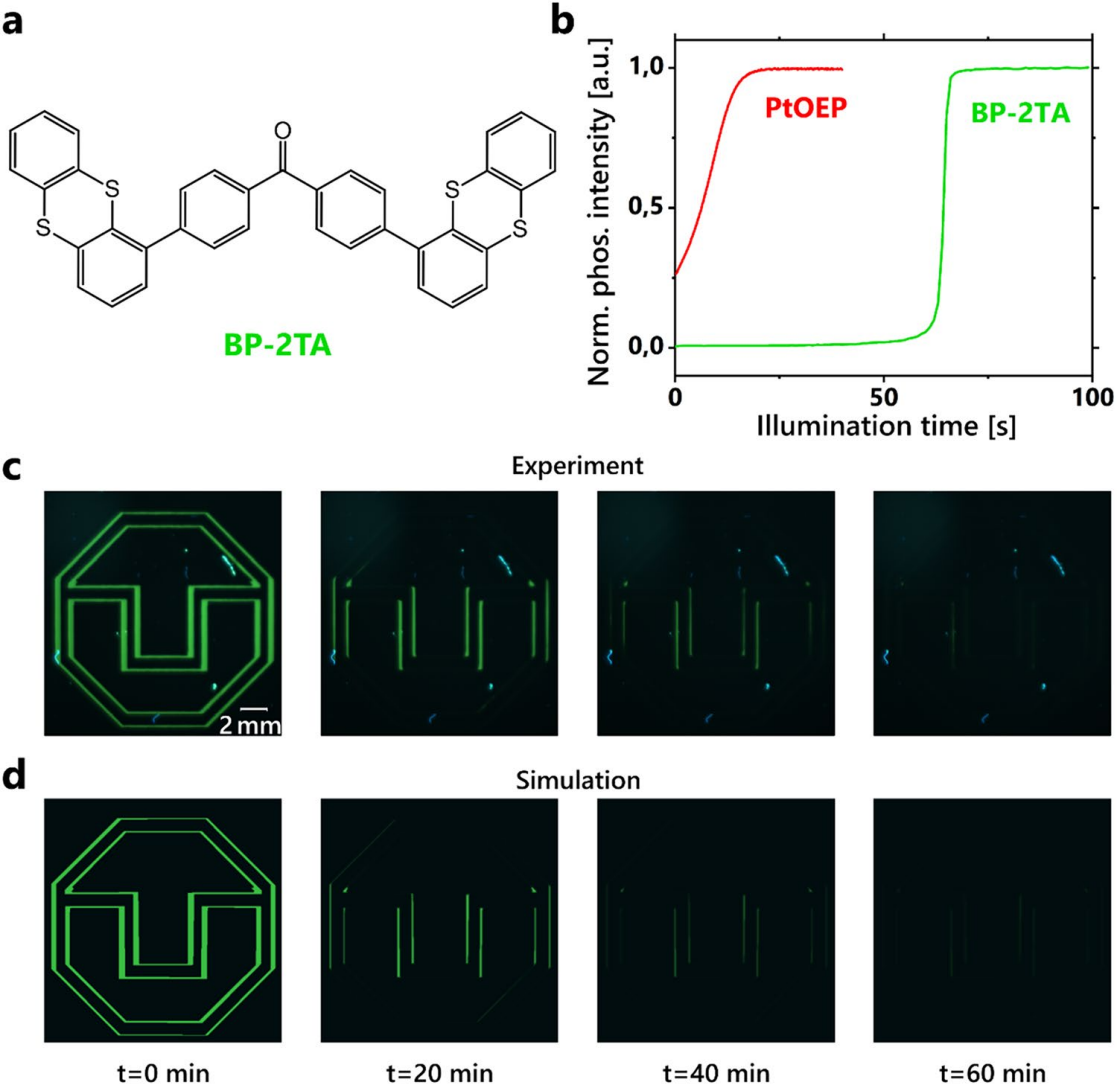
(**a**) We transferred the knowledge to a system used in PLTs, which is based on the emitter BP-2TA. The host system remains unchanged. (**b**) The normalized activation profile shows a delayed activation, steeper slope and higher on–off-ratio for BP-2TA compared to PtOEP. (**c**) The series of images shows the temporal evolution of a pattern (TU Dresden logo) inscribed in a PLT. The emission layer of the PLT consists of PS35k as host, doped with 5 wt% of BP-2TA. The pattern disappears within an hour due to lateral oxygen diffusion. The thinnest lines of the logo are already no longer visible after 20 min. (**d**) Shown here is a 2D simulation of the PLT pictured above. The diffusion coefficient *D* = 0.13 × 10^–7^ cm^2^/s best reproduces the real recorded images.

PtOEP is an excellent dye for the purpose of oxygen sensing and the respective diffusion measurements. Given other application scenarios such as luminescent labelling, a more pronounced on–off-ratio as well as different color, quantum yield, turn-on-illumination or doping ratio are required^20,21^. Suitable properties for labelling applications can be found, e.g., in 4,4′-dithianthrene-1-yl-benzophenone (BP-2TA), see Fig. 5a. A comparison reveals that the system based on BP-2TA requires a longer illumination time to be activated but yields a higher on–off-ratio as well as a steeper turn-on-slope as it is shown in Fig. 5b. Consequently, the readout contrast for labelling applications based on BP-2TA outcompetes the PtOEP quite significantly. For this demonstration purpose all processing parameters as well as the host system PS35k remained unchanged. Only the dye and its concentration were changed from PtOEP (0.67 wt%) to BP-2TA (5 wt%).

Finally, we transferred our findings to perform a stability test of a labelling application using BP-2TA. In this scenario, a two-dimensional pattern (TU Dresden logo) was inscribed into the programmable luminescent tag. The pattern vanishes over time since the diffusing oxygen quenches the luminescence in the activated areas.

The diffusion measurement result for PS35k (*D* ≈ 0.8 × 10^–7^ cm^2^/s) was used to attempt to predict the temporal evolution of information written into a PLT. After imprinting the information, a picture was taken every 20 min, resulting in the image series shown in Fig. 5c. A 2D simulation that best replicates the recorded images uses *D* = 0.13 × 10^–7^ cm^2^/s and is depicted in Fig. 5d. The decrease of the diffusion coefficient can be explained by the higher proportion of the small BP-2TA molecules in the considered system^28^. Hereby we presume that, alongside with the polymer chain length and processing conditions, also the emitter concentration influences *D*.

## Discussion

In summary, this article presents a new and contact-free method to determine the oxygen diffusion coefficient *D* in bulk polymer glasses exploiting the quenching of organic room-temperature phosphorescence by oxygen. Consequently, the values we determine do not suffer from any sorption–desorption surface effects. Instead of employing heavy equipment, such as gas mixing or vacuum equipment, our method is based entirely on camera-recorded images of a PLT. This allows us to track the spatial and temporal evolution of the phosphorescence intensity, which can then be translated to the oxygen concentration in the film in units of (*k*_q_*τ*_0_)^−1^ using the Stern–Volmer equation. Through the use of a stripe pattern, the data can be reduced to one spatial dimension, further simplifying the analysis. Applying a fitting routine and solving the oxygen evolution over time with a heat differential equation, the oxygen diffusion coefficient *D* can be calculated.

We see possible applications of the method described here, for example, in a study of oxygen diffusion as a function of fabrication process and material parameters of thin polymer films, i.e. dopant concentrations, polymer chain length, annealing temperature and duration, or the coating process. Also, the storage duration of information inscribed in a PLT can be investigated if the same material system and processing is used.

For the purpose of this study, the dye PtOEP seemed to be well-chosen since it allowed to discriminate the relative oxygen concentration over a large number of timesteps. As it can be obtained from Fig. 5b, it still provides a low relative phosphorescence intensity at maximum oxygen saturation, but shows a significantly less step-like behaviour compared to BP-2TA. This is due to its triplet state lifetime of about 50 microseconds compared to 30 ms for BP-2TA^21^. In turn, dyes showing an even lower triplet state lifetime yield higher relative intensities at maximum concentration according to the Stern–Volmer equation. Hence, a simple CCD camera pixel would not be sufficient for the experiment anymore, given the increasing signal-to-noise ratio.

Additionally, beta-carotene appears to be effectively protecting the dye from degradation. As in many biological systems the underlying mechanisms are expected to be both singlet oxygen quenching and the formation of endoperoxides in beta carotene (C). The oxygen quenching may be the more prominent process and is generally described as^38^

$${}^{1}{{\text{O}}}_{2} + {\text{C}} \to { }^{3}{\text{C}} +{ }^{3}{{\text{O}}}_{2}$$

$${}^{3}{\text{C}} \to {\text{C}} + {\text{heat}}.$$

In contrast, endoperoxide formation leads to consumption of the original carotenoid and the subsequent loss of its quenching functionality^39^. It is also the suggested trapping mechanism in the polystyrene host matrix.

Autooxidation of beta-carotene by ^3^O_2_ is a third but scarce process discussed in literature. In our experiment, it must necessarily lead to activation of the samples over time. However, this was not observable for any sample.

Regarding the limitations of the method, we found several points that need to be improved in future studies. The scaling factor *F* can be eliminated by using a homogeneous excitation profile, resulting in *D* being the only fitting parameter. Taking into account the sublinear behaviour of the Stern–Volmer relationship as it can be found in comprehensive literature rather than the idealized linearization may further improve the accuracy of the fitting^36^. Uniform samples without defects increase the evaluable area and reduce scattering. Defects on the sample tend to occur either as saturated intensity values due to scattering or as unusually low intensity values due to holes in the oxygen barrier layer. Scattered light also distorts the recorded intensity values in these areas, thereby manipulating the photoconsumption and oxygen concentration values. Furthermore, with uniform samples, periodic boundary conditions could be used in the simulation, rather than using the experimental data as boundary. Increasing the color depth of the camera from 8-bit to e.g. 16-bit improves the resolution per channel by a factor of 256, which would reveal smaller concentration changes. By employing a camera with increased sensitivity, systems with a smaller amount of emitter can be studied, as the illumination time per image could stay the same or even decrease. This would yield results closer to a pure, undoped polymer film and reduce the amount of photoconsumption during the measurement. The use of stripe patterns with smaller width and a high-resolution camera reduces the measurement time, as it allows to observe the oxygen diffusion on a smaller scale. Last, the time steps between two images during the diffusion phase also need optimization to find the best balance between photoconsumption and the number of support points for the simulation.

After demonstrating the general measurement principle, further research could exploit the proposed method for different polymer types and varying chain length. In this regard, the solubility of polymer, dye and β-carotene as well as transparency and processability may set a limit regarding the expansion of this method.

The structural changes during the annealing and photoconsumption processes should also be analyzed to better understand their influence on the diffusion coefficient. Earlier studies already provide initial indications with respect to the effect of photoconsumption for various polymers in similar systems^37^.

## Methods

### Experimental part

#### Sample preparation

The emissive layers were spin coated with 16 rps over 60 s (ramp 3 s) on a 1 × 1 in. quartz glass substrate. We used solutions with 100–300 mg/ml of the respective polymer (PS13k: 41928, Alfa Aesar; PS35k: 331651, Sigma-Aldrich; PS192k: 430102, Sigma-Aldrich; PS280k: 182427, Sigma-Aldrich; PS350k: 441147, Sigma-Aldrich), 0.3 mg/ml PtOEP (PtO534, Frontier Scientific) and 0.3 mg/ml beta-carotene (C9750, Sigma-Aldrich) dissolved under stirring on a hotplate at 75 °C in a mixture of ethanol and toluene with a 1:5 ratio. The resulting films have a thickness of about 2–9 µm. Heating for 15 min at 115 °C evaporates the remaining solvent and ensures that the film converts to the glassy phase by overcoming the polystyrene glass transition temperature of about 100–107 °C^33^. The solution for the encapsulation consists of Exceval™ (AQ-4104, Kuraray) diluted in deionised water at a concentration of 100 mg/ml. Before use, it is stirred at 95 °C for 30 min, followed by a cooling phase to room-temperature. We added the encapsulation layer by drop casting 350 µl of the solution onto the emission layer and letting it dry for 12 h in the dark.

#### Setup

The excitation part of the experimental setup consists of a high power 365 nm LED (M365LP1, Thorlabs), equipped with a collimating lens (ACL25416U-A, Thorlabs), a square diffuser (ED1-S20-MD, Thorlabs) and a 370 ± 18 nm bandpass filter (86-982, Edmund Optics) to achieve a homogeneous rectangular illumination at the sample position. In addition, the bandpass filter is tilted by 10° with respect to the sample to prevent reflections of the sample in the filter being recorded.

We use an Arduino microcontroller to trigger the LED with pulses of 10–40 ms. We constructed a sample holder, with the option of placing a shadow mask directly in front of the sample, plus an additional piece of cleanroom paper to serve as a fluorescent screen for tracking the LED intensity. The detection part of the setup is a CCD camera (acA1920-40uc, Basler AG) with focusing lenses (HF25XA-5M, Fujifilm) and a 450 nm longpass filter (FELH0450, Thorlabs). Again, the filter is tilted by 10° with respect to the sample to prevent reflected UV light from the LED to hit the sample again from the back side and other reflections on the sample from being recorded. The setup is controlled using the SweepMe! measurement software^34^.

The camera records the RGB values as integers in the range of 0–255 per pixel and color channel, corresponding to a color depth of 8-bit. In order to linearly correlate the color value to the intensity of each recorded image, daylight corrections such as the gamma correction factor and the balance ratio are set to the value of 1.

The polystyrene-embedded emitter PtOEP shows an emission peak at around 650 nm and no emission below 600 nm under excitation at 365 nm. Consequently, only the red color channel with a spatial resolution of 960 by 600 pixels is used to resolve the emission intensity. In contrast, we use the blue and green channels to monitor the fluorescence of the LED intensity tracker.

#### Measurement routine

Before starting the actual measurement, we used a similarly prepared sample to determine the optimal exposure time *t*_exp_ and to estimate the number of pulses needed to activate the sample. The measurement routine executed by SweepMe! consists of three phases, which we followed consecutively. First of all, we illuminated the sample through a striped shadow mask (negative profile see Fig. 3a) to achieve a spatial oxygen concentration gradient via photoconsumption (activation phase). Therefore, typically 2000–4000 LED pulses of 10–40 ms exposure time *t*_exp_ were necessary to fully activate the sample at the illuminated spots, resulting in a total exposure time of about 20–160 s. Additionally, the camera records every 50th or 100th pulse, depending on the total number of pulses.

In the second phase the shadow mask is removed and an image is recorded every 12 s (*t*_step_ ≈ 12 s) over a period of up to 8 h in order to monitor the oxygen diffusion process (diffusion phase).

The third and final phase (reactivation phase) follows the same scheme as the activation phase, but without applying the shadow mask in order to remove any remaining oxygen in the sample and to obtain an image of the maximum spatial intensity distribution over the entire sample surface (normalization image).

The size of the pixels (about 62 µm per red pixel) was determined from the known width and distance of the stripes from the shadow mask layout.

#### Film thickness measurement

The thickness of a film was determined using a Veeco Dektak 150 Profilometer.

### Data analysis

#### Reference activation curve

The reference activation curve is extracted from the images taken during the activation phase. Therefore, all images of this phase are normalized to the normalization image and their respective LED intensity. To also correct for the different activation doses, caused by the varying LED intensity, the excitation time of each image must be multiplied by its LED intensity.

Then the red pixel values in a homogeneous spot are averaged and saved (cf. Fig. 3a, spot 1). This results in a normalized activation curve *I*_act_(*t*) that can be used as a reference response of the system to a certain illumination time *t* with an unknown UV intensity (or a certain UV dose with a known UV intensity).

#### Preprocessing

First, the LED intensity *I*_LED_(*n*) of all *n* images taken during the diffusion phase is saved for later calculation of the photoconsumption. The normalization image *n*_N_ serves as standard for the reference LED intensity *I*_LED_(*n*_N_), as well as the maximum achievable red color value at each pixel. In order to account for temporal fluctuations of the LED intensity, the red color values of each pixel in each image *I*(*x,y,n*) are normalized according to the ratio of the current and the standard LED intensity of the normalization image. Furthermore, we normalized the red values of each image according to the ratio of the current and the normalization image. This is to compensate for variations in the emission layer thickness and small spatial inhomogeneities of the LED intensity. This complete normalization process is performed using the following formula

3 $${{I}_{\text{norm}}(x,y,n)=\frac{I(x,y,n) {I}_{\text{LED}}\left({n}_{\text{N}}\right)}{I\left(x,y,{n}_{\text{N}}\right) {I}_{\text{LED}}(n)},}$$

where *I*_norm_(*x,y,n*) is the normalized red pixel value with *x* and *y* the pixel position and *n* the image number, *I*(*x,y,n*) the initial recorded red pixel value, *I*_LED_(*n*_N_) the LED intensity of the normalization image, *I*(*x,y,n*_N_) the red pixel value of the normalization image at (*x,y*), and *I*_LED_(*n*) the LED intensity of image *n*.

Only areas without visible defects within one stripe pattern are used for further evaluation (cf. Fig. 3a, spots 2–5). The normalized red pixel values in these areas are averaged along the axis of the pattern, reducing the data by one spatial dimension. Thus, each diffusion measurement results in six preprocessed data sets. Namely the reference activation curve *I*_act_(*t*), the LED intensity during the diffusion phase *I*_LED_(*n*) and four data sets containing the intensity progression of the pattern *I*_diff_(*x,n*), hereafter referred to as diffusion curves.

#### Calculate photoconsumption

The photoconsumption during the measurement plays an important role, as the system is inevitably illuminated by UV light each time an image is taken during the diffusion phase, thus removing some of the remaining oxygen.

To account for this activation during the diffusion phase, the amount of oxygen removed from the system during each image acquisition must be calculated, resulting in two sets of data containing the oxygen concentration immediately before [*Q*(*x,n*)]_pre_ and after [*Q*(*x,n*)]_post_ each image.

The calculation is performed for each value *I*_diff_(*x,n*) of the diffusion curves in the following way. First, the value is mapped onto the interpolated activation curve *I*_act_(*t*), resulting in a time *t*_act_ corresponding to the initial illumination time during the activation process. Then, the times representing the start *t*_*n*,pre_ and the end *t*_*n*,post_ of each image acquisition are calculated:

4 $${t}_{n,\text{pre}}={t}_{\text{act}}-{I}_{\text{LED}}\left(n\right)\frac{{t}_{\text{exp}}}{2},$$

5 $${t}_{n,\text{post}}={t}_{\text{act}}+{I}_{\text{LED}}\left(n\right)\frac{{t}_{\text{exp}}}{2}.$$

The additional multiplication by *I*_LED_(*n*) corrects the exposure time *t*_exp_ for varying LED intensities during the diffusion phase. Now, the intensity at the beginning *I*_diff,pre_(*x,n*) and at the end *I*_diff,post_(*x,n*) of each image acquisition can be determined from the activation curve

6 $${I}_{\text{diff},\text{pre}}(x,n)={I}_{\text{act}}\left({t}_{n,\text{pre}}\right)$$

7 $${I}_{\text{diff},\text{post}}(x,n)={I}_{\text{act}}\left({t}_{n,\text{post}}\right)$$

Finally, the oxygen concentration at these intensities is calculated using the Stern–Volmer equation (Eq. 1). Since the intensity is now unitless after normalization (Eq. 3) and the maximum intensity *I*([*Q*] = 0) is equal to 1, the rearranged Stern–Volmer equation simplifies to

8 $${\left[Q\left(x,n\right)\right]}_{\text{pre}}=\left(\frac{1}{{I}_{\text{diff},\text{pre}}(x,n)}-1\right)\frac{1}{{k}_{\text{q}}{\tau }_{0}},$$

9 $${\left[Q\left(x,n\right)\right]}_{\text{post}}=\left(\frac{1}{{I}_{\text{diff},\text{post}}(x,n)}-1\right)\frac{1}{{k}_{\text{q}}{\tau }_{0}}.$$

To determine the diffusion coefficient *D*, it is sufficient to express the oxygen concentration [*Q*] in units of (*k*_q_τ_0_)^-1^, which further simplifies Eqs. (8) and (9). In addition, *k*_q_ and τ_0_ do not need to be determined separately. After receiving [*Q*(*x,n*)]_pre_ and [*Q*(*x,n*)]_post_, the data processing is complete.

#### Fitting the diffusion coefficient

When we looked at the acquired data, we immediately noticed an unknown source of oxygen in the oxygen-depleted areas of the pattern. This source is most active during the first 30 min of the diffusion phase. Here we suspect slow oxygen diffusion from the barrier layer into the emission layer, since the total amount of new oxygen is small compared to the saturation concentration of the emission layer (about 2%). The process is also much slower compared to lateral diffusion within the emission layer, where a 2 µm thin oxygen-free region would refill by about 10% in less than one second. This is consistent with the low saturation concentration and small diffusion coefficient of the oxygen barrier layer.

However, in order to keep the fitting routine a one-dimensional problem, we decided to skip the first 30 min of the diffusion phase (150 time steps) and use the oxygen concentration distribution [*Q*(*x*,*n* = 150)]_post_ as the initial condition for the simulation. The boundary conditions are taken from the measured data. We stop the simulation after minute 120 of the diffusion phase (*n* = 600), since no significant oxygen gradient is visible from this point on.

The fitting routine solves Eq. (2) using the forward Euler method with one time step (n = 1, *t*_step_ ≈ 12 s) divided into 5 intermediate steps ($\Delta n$ = 1/5) to ensure numerical stability:

10 $$[Q\left(x,n+\Delta n\right){]}_{\text{sim}}=\left[Q\left(x,n\right)\right]+D\cdot \Delta n\frac{{\partial }^{2}}{\partial {x}^{2}}\left[Q\left(x,n\right)\right].$$

This simulates the diffusion process from the oxygen distribution after one image [*Q*(*x*,*n*)]_post_ to the oxygen distribution before the next image [*Q*(*x*,*n* + 1)]_pre_. Then, the calculated photoconsumption during the next image acquisition is subtracted from the simulated data using

11 $$[Q(x,n+1){]}_{\text{sim},\text{post}}=[Q(x,n+1){]}_{\text{sim}}-F\cdot \left([Q(x,n+1){]}_{\text{pre}}-[Q(x,n+1){]}_{\text{post}}\right)$$

with the photoconsumption scaling factor *F*, which corrects for spatial LED intensity variations over the sample. The simulation treats the photoconsumption process as instantaneous, which we assume to be a valid assumption due to the short exposure time compared to a diffusion time step (10–40 ms ≪ 12 s). This results in the concentration distribution [*Q*(*x*,*n* + 1)]_sim,post_, which serves as the starting point for the next diffusion step. Finally, the fitting routine adjusts the parameters *D* and *F* until the weighted residual

12 $$\Delta Q={\sum }_{x}{\sum }_{n}\frac{\left|[Q(x,n){]}_{\text{post}}-[Q(x,n){]}_{\text{sim},\text{post}}\right|}{\sqrt{[Q(x,n){]}_{\text{sim},\text{post}}}}$$

is minimized. The iterative optimization was performed using the Python library *lmfit*^35^. The resulting diffusion coefficient is in units of $\frac{{\text{px}}^{2}}{{t}_{\text{step}}}$, which is then converted using the determined conversion factors of about $\frac{62\,\upmu \text{m}}{\text{px}}$ and $\frac{12\,\text{s}}{{t}_{\text{step}}}$.

## Data Availability

The full data is available from the corresponding author upon reasonable request.
